# Screening of microbial consortium with high efficiency of lignin-degrading and its synergistic metabolic mechanism

**DOI:** 10.3389/fmicb.2025.1709019

**Published:** 2025-10-31

**Authors:** Jinchuang Ru, Zhiyang Jiang, Jiayu Li, Xiang Li, Zhencheng Su, Tong Li, Mingkai Xu

**Affiliations:** ^1^Institute of Applied Ecology, Chinese Academy of Sciences, Shenyang, China; ^2^University of Chinese Academy of Sciences, Beijing, China; ^3^Shenyang Pharmaceutical University, Shenyang, China

**Keywords:** lignin degradation, microbial consortia, degradation pathway, metagenomics, metabolomics

## Abstract

**Introduction:**

Lignin is difficult to degrade, which makes its high-value utilization a challenge. So finding an efficient method to degrade lignin is very important. At present, microbial degradation is considered to be one of the most effective and environmentally friendly degradation methods that is widely accepted.

**Methods:**

This study enriched three lignin-degrading microbial consortia R0, R1 and R2 using alkali lignin as the sole carbon source under 15 °C conditions. Using the methods of 16S rRNA sequencing, metagenomics, and metabolomics, the degradation mechanism of these three microbial consortia were systematically analyzed.

**Results:**

The microbial consortium R0, which has the best degradation efficiency, can degrade more than 80% within 6 days, with dominant genera being *Achromobacter* and *Pseudomonas*. The dominant genera in other two microbial consortia R1 and R2 are *Pseudomonas* and *Achromobacter* in R1, *Pseudomonas* and *Sphingobacterium* in R2. Protocatechuic acid is a central intermediate in the degradation of lignin, its degradation pathway was fully annotated in microbial consortia R0 and R1. Microbial consortium R0 has the most abundant of AA (Auxiliary Activities) family genes annotated as carbohydrate annotation enzymes. The dominant genera in the microbial consortium R0 based on AA family gene abundance were *Pseudomonas* and *Achromobacter*.

**Discussion:**

Our results indicated that *Pseudomonas* is the dominant genus in lignin degradation, the metabolic potential of other abundant genera suggests a possible complementary role in the lignin degradation process. In the lignin degradation system with *Pseudomonas* as the dominant genera, the degradation of protocatechuic acid is the core of the degradation process. This study could enrich the mechanism of efficient and stable lignin degradation by microbial consortium, and could provide theoretical guidance for the development of lignin biodegradation technology in industry.

## 1 Introduction

Lignin, a polymer rich in aromatic compounds and the mainly structural component of plants, is considered the most abundant and renewable source of aromatic carbon on the planet (Wang et al., 2024). Lignin is known to be the second most inexhaustible natural organic polymer, and its depolymerization products includes polyhydroxyalkanoates, 4-hydroxybenzaldehyde, vanillyl alcohol, cis-viscoconate, 2-pyrone-4,6-dicarboxylic acid and syringyl alcohol, etc. (Ponnusamy et al., 2019). These depolymerization products of lignin can be further converted into many raw material products for chemical industry. For example, 4-hydroxybenzyl alcohol, vanillyl alcohol, and syringyl alcohol can be converted by microorganisms into lipid biosynthesis (Liu et al., 2022). People are committed to exploring the technology for depolymerization, transformation and value-added utilization of lignin.

Lignin has a complex and recalcitrant structure consisting of three major phenylpropanoid monomer subunits: syringyl (S), guaiacyl (G), and *p*-hydroxyphenyl (H) (Upton and Kasko, 2016). These subunits are interconnected by various carbon–carbon and carbon–oxygen bonds such as *β*-O-4 and *α*-O-4 and have different functional groups including methoxy, carbonyl, hydroxyl, and carboxyl groups (Balestrini et al., 2024). However, the heterogeneity and macromolecular nature of lignin pose significant challenges for its efficient utilization. Therefore, efficient lignin degradation is one of the most critical steps to break down the physical barrier and release renewable resources for biofuels, bioplastics, and other value-added products.

The efficient depolymerization of lignin has always been a research topic in the field of high-quality utilization of lignin. The depolymerization efficiency of lignin affects the lignin bioconversion process and ultimately the viability of the depolymerization products for industrial applications (Balestrini et al., 2024). Various physical and chemical methods have been extensively studied, including thermochemical, electrochemical, and photocatalytic depolymerization of lignin (Pei et al., 2024). Moreover, biological depolymerization of lignin performed by specific microorganisms offers advantages such as lower energy consumption, milder reaction conditions, and reduced carbon emissions, which has become the most promising lignin depolymerization method for industrial application. Therefore, it has attracted the most attention from scientists and engineers.

Many fungi and bacteria in nature have the ability to degrade lignin by producing different types of lignin-degrading enzymes. For example, white-rot fungi and brown-rot fungi can secret various peroxidase and hydrolases to disrupt the stubborn structure of lignin. However, the slow growth of fungi and their enzyme activity are highly susceptible to environmental interference, which limits their industrial-scale application (Eastwood et al., 2011). In contrast, bacteria possess superior environmental tolerance, enabling lignin degradation across extreme conditions including thermal extremes. Specifically, natural enrichment processes without fungal inhibitors at 15 °C and 30 °C have been reported to yield exclusively bacterial consortia capable of effective degradation, some bacteria retaining this capability even at temperatures as low as 5 °C, which make bacteria more suitable for applications (Poyntner et al., 2021; Yadav et al., 2023).

According to reports, a single bacterium strain has a limited ability to cleave all the chemical bonds in lignin. In contrast, the microbial consortia have shown more advantages in the biotransformation of lignin (Hu et al., 2025). In nature, The biodegradation of lignin is typically accomplished by a variety of microorganisms, forming a specific degrading microbial consortium in which each member performs a specific subfunction (Wilhelm et al., 2019). A variety of microorganisms exercise the complete degradation of complex compounds through symbiotic and synergistic metabolic networks. Metabolites of one group of microorganisms may be the metabolic substrates of another group of microorganisms (Meng et al., 2022). Therefore, in practical application, the effect of using microbial consortium is much greater than that of a single bacteria (Ali et al., 2017). Research on the metabolic mechanism of microbial consortium can help in the effective use of microbial consortium. The application of “omics” methods to identify the metabolic function of multi-species complexes is well-established and credible (Hess et al., 2011). With the rapid development of multi-omics technology, scientific issues such as the dynamic changes in community structure, the mechanisms of symbiosis and synergistic metabolism among different microorganisms, and the metabolic pathways of complex substrates during the degradation of specific substrates have been systematically revealed (Hu et al., 2025). This has important scientific significance for constructing artificial combined microbial consortium to achieve efficient and complete degradation of lignin under harsh industrial conditions.

In this study, alkali lignin was used as a sole carbon source enrich the lignin-degrading microbial consortia, using a “top-down” synthetic functional microbiome strategy. Three efficient lignin-degrading microbial consortia were obtained through enrichment and subculturing from rice straw compost, corn straw compost, and soil from corn straw return. 16S rRNA sequencing, metagenomics and metabolomics techniques were used to analyze the community dynamics of the microbial consortia during the degradation process, predict the main functional groups and key degradation genes, and to analyze the mechanism of their symbiotic metabolism, so as to provide theoretical guidance for the construction of artificially degraded microbial consortia.

## 2 Materials and methods

### 2.1 Acquisition of materials and enrichment

In this experiment, three lignin-degrading microbial consortia (R0, R1, and R2) were obtained through continuous enrichment from distinct sources sampled at a depth of 10–20 cm: R0 from composted rice straw in Changsha City, Hunan Province; R1 from farmland soil with a five-year history of maize straw returning in Fuxin City, Liaoning Province; and R2 from composted maize straw in Shenyang City, Liaoning Province. The formulation of lignin medium for consortia enrichment contains 2 g/L (NH_4_)_2_SO_4_, 2 g/L NaNO_3_, 1 g/L K_2_HPO_4_, 1 g/L NaH_2_PO_4_, 1.5 g/L MgSO_4_, and 5 g/L alkaline lignin. All the samples were autoclaved at 121 °C for 20 min before inoculation. To enrichment culturing, the stalk treatment for microbial consortia R0 and R2 were the same, the stalk was cut with scissors to a length of 1–1.5 cm, take 5 g of stalk and soak it in 50 mL of distilled water for 20 min. The mixture was subsequently vortexed for 2 min to dislodge the microbes, and 1 mL of the supernatant was added to 100 mL enrichment medium. Take 5 g of stalk returned soil sample into 45 mL distilled water for 20 min. The mixture was vortexed thoroughly for 2 min to homogenize the suspension. The solution was then serially diluted from 10^−1^ to 10^−5^ g/mL, take 1 mL of 10^−5^ dilution and add it into 100 mL enrichment medium. Lignin degradation rate was measured every 2 days until the eighth day. Take 1 mL of culture and add it to 100 mL of culture medium for the next round of subculture, continuing for more than five times in total. Lignin degradation experiments were conducted using the same 5 g/L alkali lignin concentration as in the enrichment medium. All cultures were incubated statically at 15 °C. Inoculations were performed with 1% (v/v) of stationary-phase pre-cultures to ensure consistent starting biomass across replicates. If the degradation rate remains stable for three consecutive generations, the enrichment is considered successful.

### 2.2 Lignin content test

In order to enrich stable and efficient lignin-degrading microbial consortium, we used alkali lignin as a sole carbon source and conducted multiple rounds of sub-culturing at 15 °C. We first passaged microbial consortia R0, R1 and R2 for more than five times according to the 8-day period, after which the degradation rate was tested for three consecutive generations, once every 2 days.

Based on the obtained microbial consortium with stable lignin degradation efficiency, a new subculturing round was performed. During this passage, the degradation rate was detected daily. Samples from the third and sixth days were selected for subsequent 16S rRNA sequencing, metagenomic analysis, and metabolomic profiling. The lignin content was detected with a lignin content detection kit (Catalog No. BC4205), Solarbio Science & Technology Co., Ltd., Beijing, China, according to the manufacturer’s instructions. Specifically, reagents from the kit were sequentially added to the samples, followed by incubation in a water bath to develop color for lignin. The lignin content was then determined using a microplate reader. The final product of the reaction was detected by a microplate reader at 280 nm.


z=(y−x)/y×100%

*y* is the lignin content of the control group, *x* is the lignin content of the experimental group, and *z* is the degradation rate. An uninoculated control (containing only the enrichment medium and lignin substrate) was included in all degradation experiments to account for any non-biological degradation. The lignin degradation rates reported throughout this study are net values.

### 2.3 16S rRNA gene and metagenomics sequencing of enriched microbial consortia

To collect the bacterial sediments, the cultures of consortia R0, R1 and R2 were sampled at the time points of day 3 and day 6, with 15 mL per sample and a total of 6 parallels. High-throughput sequencing was performed by Sangon Bioengineering (Shanghai) Co., Ltd. to detect the abundance and diversity of microorganisms and related genes in the microbial consortium.

The V3-V4 region of the 16S rRNA gene from four microscopic worlds was amplified using universal bacterial primers 341F (5’-CCTACGGGNGGCWGCAG-3′) and 805R (5’-GACTACH VGGGTATCTAATCC-3′). The final library was sequenced on the Illumina® MiSeq™ platform. Details of high throughput sequencing analysis can be found in Sangon Biotech ™ Service website. Illumina Miseq™/Hiseq™ raw image data files are converted into Sequenced Reads, which we call Raw Data or Raw Reads, and the results are stored in the FASTQ (fq) file format, which contains the sequence information of the sequenced sequences (reads) and their corresponding sequencing quality information. The data obtained from off-machine sequencing is paired-end sequence data, which contains barcode sequences as well as primer and adapter sequences added during sequencing. First, the primer and adapter sequences need to be removed. Then, based on the overlap relationship between the PE reads, the paired reads are merged into a single sequence. After that, the samples are identified and distinguished according to the barcode sequences to obtain the data for each sample. Finally, quality control filtering is performed on the data of each sample to obtain the effective data for each sample.

For metagenomics sequencing, prepare libraries with insert fragments of about 500 bp in length, with an initial total of about 500 ng of DNA. Dilute the DNA to 130 μL using Elution Buffer in a 0.5 mL Covaris DNA fragmentation tube. The fragmented DNA fragments were concentrated and recovered using 1x Hieff NGS™ DNA Selection Beads. Purified and detected using Qubit 4.0. Library size was detected by 2% agarose gel electrophoresis, and library concentration was determined using a Qubit 4.0 fluorescence quantifier, with all samples mixed in 1:1 aliquots. The splicing software Megahit (version V 1.2.9), which is based on the principle of De Bruijn graph, was used to perform multi-sample hybrid splicing of clean reads. After that, the clean reads of each sample were compared with the assembled contigs, and the unmapped PE reads were obtained. SPAdes software (version V 3.13) was used to continue hybrid splicing of the unmapped reads, for the contigs generated from the two splicing assemblies, sequences smaller than 500 bp were filtered. After that, the clean reads of each sample were aligned to the assembled contigs using bowtie2 software (version V2.1.0), and the unmapped PE reads were obtained, the unmatched reads were then spliced into the assembled contigs using SPAdes software (version V3.13), and the contigs were filtered to sequences smaller than 500 bp, and then analyzed downstream by statistics and subsequent gene prediction. The CAZy annotation was conducted using HMMER3 against the CAZy database (E-value < 1e-5). The abundance of Auxiliary Activity (AA) families was statistically analyzed based on these CAZy annotations, by summing the TPM-normalized read counts of all constituent genes annotated as AA families. A descriptive analysis of KEGG pathway distributions was performed based on gene counts. Pathways containing the highest number of genes relevant to lignin and aromatic compound degradation were identified as the key functional categories, providing an overview of the predominant metabolic potential in the consortia. Differentially abundant genes were identified using a Student’s t-test comparing gene abundance between Day 3 and Day 6. Genes with a *p*-value < 0.05 were considered significant and used for subsequent pathway analysis. The sequencing data generated in this study have been deposited in the NCBI Sequence Read Archive (SRA) under the BioProject accession number PRJNA1327111.

### 2.4 Metabolomic analysis

To detect changes in metabolites during lignin degradation, the cultures of consortia R0, R1 and R2 were sampled at the time points of day 3 and day 6, each sample was 2 mL, a total of 6 parallels. The obtained samples were stored at −80 °C. 100 μL of the sample was added to the corresponding numbered 1.5 mL centrifuge tube, and 100 μL of 70% methanol containing internal standard extract (less than 100 μL, the extract was added in a ratio of 1:1 (V/V)), the internal standard extract was prepared as 1 mg of standard dissolved in 1 mL of 70% methanol water to prepare 1,000 μg/mL standard mother liquor, and 1,000 μg/mL mother liquor was further diluted with 70% methanol to prepare 250 μg/mL internal standard solution. Vortex for 15 min, 12,000 r/min, 4 °C, and centrifuge for 3 min. The supernatant was pipetted, filtered in a microporous filter (0.22 μm pore size), and stored in a vial for LC–MS/MS detection. Samples were tested by Sangon Bioengineering (Shanghai) Co., Ltd. For non-target LC–MS metabolomics analysis. Metabolites were identified by querying the laboratory’s own database, integrating public databases, predictive libraries, and the metDNA method. Finally, substances with a cumulative score greater than 0.5 and a CV value less than 0.5 in the QC samples were extracted and identified, and positive and negative modes were merged (retaining substances with the highest qualitative grade and the smallest CV value) to generate an Excel format data file for downstream analysis. Differential metabolites were identified based on a threshold of *p* < 0.05 and VIP ≥ 1.

### 2.5 Statistical analysis

The lignin content was detected in three parallel analyses, and the rest of the analyses were six parallel. Degradation curve plotting was done using GraphPad Prism 8.0. The richness and diversity of samples were calculated with alpha diversity, including Shannon and Simpson. Principal component analysis (PCA) was used to evaluate the microbial community structure according to Bray-Curtis distance. The plotting of omics data is completed using a plotting platform. Data management and statistical analyses were performed using SPSS 25.0 software. A *p* value <0.05 was considered to be statistically significant. Networks were explored and visualized with the interactive platform gephi.

## 3 Results and discussion

### 3.1 Detection of the degradation rate of enriched bacteria

**Figure 1:**
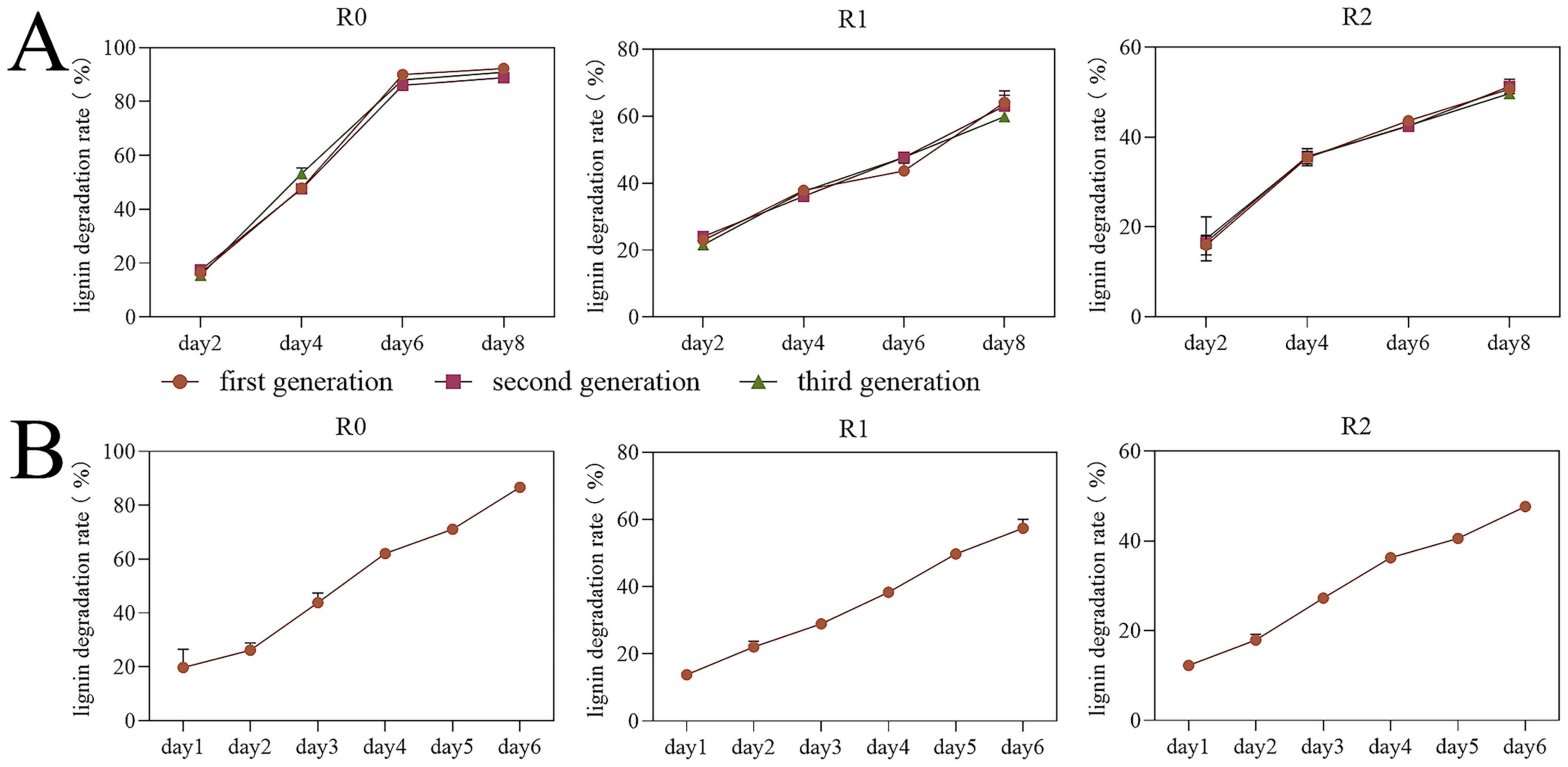
Detection results of lignin degradation rates of three microbial consortia. Net Lignin Degradation by Microbial Consortia R0, R1, and R2. The degradation rates represent net values after subtraction of the abiotic degradation observed in uninoculated control. **(A)** The results of lignin degradation rate testing across three consecutive generations. **(B)** The results of lignin degradation rate for the sample group.

The results of lignin degradation rate found that the three enriched microbial consortia R0, R1, and R2 maintained stable degradation rates for three consecutive generations. The degradation rate of microbial consortia R0, R1 and R2 could be stabilized at over 86, 57 and 47% in day 6 even 15 °C, respectively, (Figures 1A,B). Enrichment at low temperatures facilitates the application of these consortia for the decomposition of straw lignin under similarly cold conditions. Sangeeta Yadav et al. (2023) identified a highly efficient lignin degrading microbial consortium that degraded 73% of lignin on the eighth day at 37 °C. Guoxiang Zheng et al. screened a parthenogenetic anaerobic lignin degrading microbial consortium named LTF-27 which degraded 12.5% of lignin in 20 days at 15 °C (Zheng et al., 2020). The lignin degradation efficiency of consortium R0 is among the highest reported for microbial consortia, particularly under low-temperature conditions.

### 3.2 Analysis of lignin degradation microbial system

**Figure 2:**
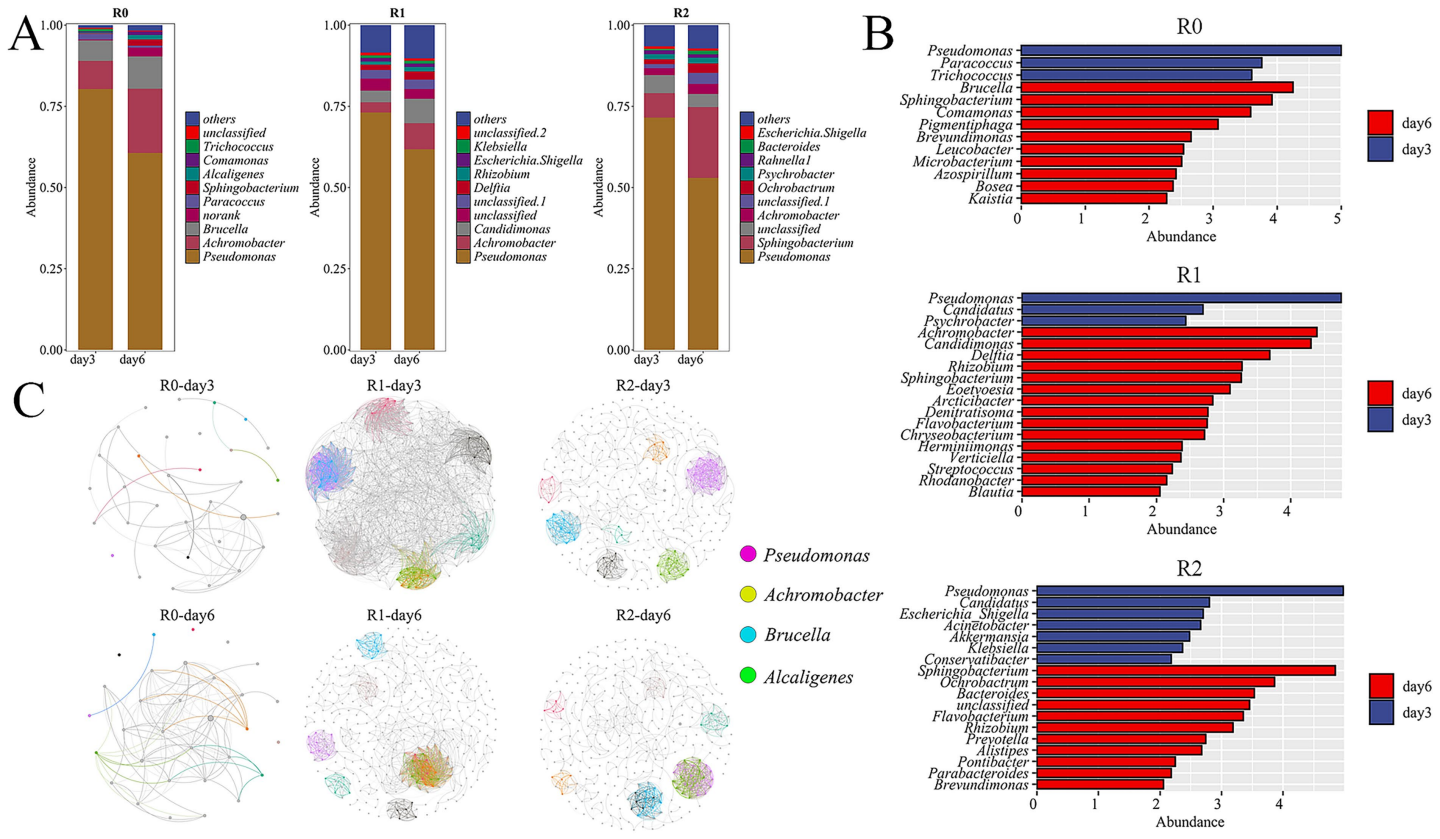
Analysis of 16S data of three microbial consortia. **(A)** Histograms of species abundance on the third and day 6 of the three microbial consortia were displayed. **(B)**microbial consortium Lefse analysis. **(C)** Microbial network diagram.

Histograms of species abundance at the genus level were plotted based on the relative sequence abundances in the 16S rRNA gene sequence library (Figure 2A). The revisions pertaining to the reviewer’s comment are located in the Results and Discussion section, specifically: The enrichment process resulted in bacterial-exclusive consortia without the application of antifungal agents. This outcome aligns with reports in the literature where bacterial consortia were dominant under similarly enrichment strategies (Xu et al., 2021). The dominant genera in R1 was consistent with that of R0, *Pseudomonas* and *Achromobacter* are the dominant genera. The relative abundance of *Pseudomonas* decreased significantly on Day 6 compared with that on the Day 3. Meanwhile, the relative abundance of *Achromobacter* increased significantly. The dominant genera in the R2 were *Pseudomonas* and *Sphingobacterium*, the relative abundance of *Pseudomonas* on Day 6 decreased significantly compared with that on the Day 3, and the relative abundance of *Sphingobacterium* bacteria increased significantly on the day 6 simultaneously. Studies have shown that *Pseudomonas*, *Achromobacter*, and *Sphingobacterium* have the capabilities to degrade lignin (Joy et al., 2019; Kamimura et al., 2019). The mutual transformation of the dominant genera under different degradation stages suggests that these genera produce and metabolize different intermediate degradation products during lignin degradation and thus facilitating the functional progression of the lignin degradation pathway. Lefse analysis counted the genera with the greatest variation in abundance. On the day 3, the genera with significant abundance differences and relative abundance ranked first in the R0, R1 and R2 microbial consortia were all *Pseudomonas*. On the day 6, among the three microbial consortia R0, R1 and R2, the genera with significant abundance differences and relative abundance ranked first were *Brucella*, *Achromobacter* and *Sphingobacterium*, respectively (Figure 2B). It is worth mentioning that the second ranked genus with significant differences in abundance on day 6 of the R0 microbial consortium was *Sphingobacterium*. This reinforces the key role of *Pseudomonas*, *Achromobacter* and *Sphingobacterium* in the lignin degradation stage.

*Pseudomonas* is known for its metabolic multifunction in different ecosystems, and *Pseudomonas* isolated from decaying wood can grow with lignin and its derivatives as the only carbon source (Peix et al., 2009; Prabhakaran et al., 2015). *Pseudomonas* was present in all of the three consortia and it became the dominant genus during the degradation process, further demonstrating that *Pseudomonas* can degrade lignin in various ecosystems. It has been shown that *Achromobacter* is also has powerful lignin degradation ability (Fang et al., 2018), which allows it to be the another dominant lignin degradation genera as *Pseudomonas*. Moreover, some studies have shown that *Sphingobacterium* can depolymerize a variety of lignin intermediate metabolites (Sohn et al., 2004; Aylward et al., 2013; Ohta et al., 2015), so it is therefore inferred that *Sphingobacterium* can degrade intermediate metabolites in the later stages of lignin degradation, which makes *Sphingobacterium* the dominant genus on the day 6 of R0, this result was also observed in the R2 microbial consortium. There are few reports on the degradation of lignin by *Brucella*, so it is speculated that the *Brucella* in R0 in our study could use intermediates of lignin to growth during the degradation process. In addition, other genera in the microbial consortia system play their respective roles in the lignin degradation process.

**Figure 3:**
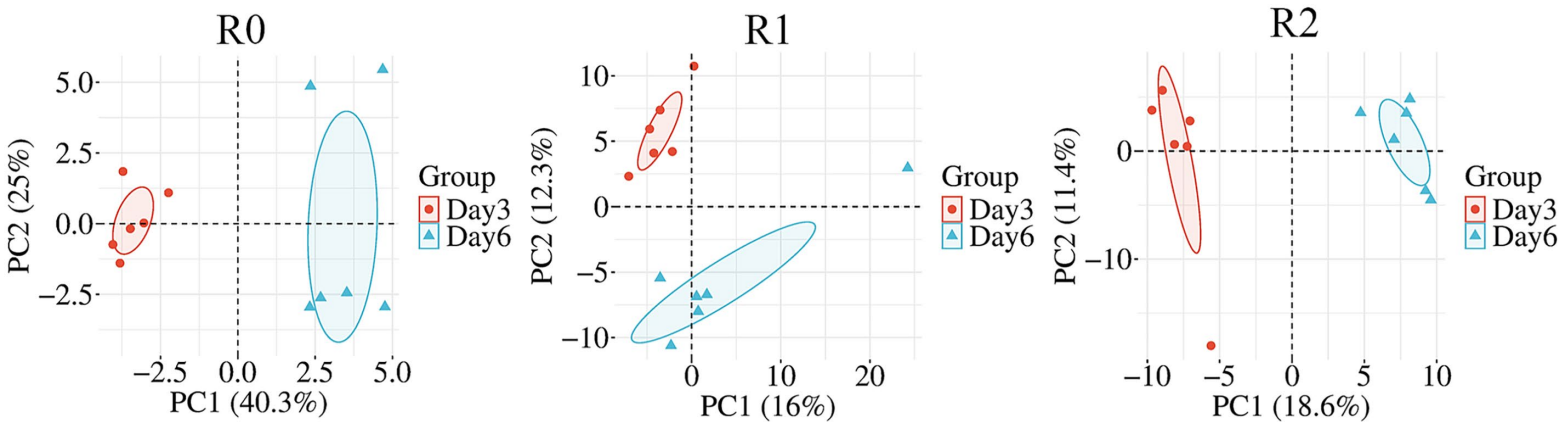
Statistical table of *β* diversity index(PCA) of three microbial consortia.

We conducted an analysis of community abundance and correlation of microorganisms in the 16S rRNA to identify key microorganisms and examine the interrelationships among the most abundant microorganisms, thereby aiding our understanding of the degradation characteristics of lignin by the three microbial consortia. In the microbial consortium R0, *Pseudomonas* was strongly negatively correlated with *Achromobacter* on both day 3 and day 6, and strongly positively correlated with *Brucella* and *Sphingomonas* on day 6. It is hypothesized that the metabolic profiles of *Pseudomonas* and *Achromobacter* are similar; however, *Pseudomonas* exhibits superior metabolic activity, The superior metabolic activity of *Pseudomonas* may influence the relative abundance of *Achromobacter* within the consortium. On the day 6, *Brucella* and *Sphingobacterium* are able to form a synergistic relationship with *Pseudomonas*, respectively, to metabolize the metabolites produced by *Pseudomonas* at a later stage in the degradation process. In the microbial consortium R1 *Pseudomonas* was strongly negatively correlated with *Achromobacter* on both day 3 and day 6. It is hypothesized that there is an inhibitory effect between the two genera, and the inhibition persisted at both time points of the assay. However, strong correlations did not exist between the dominant genera in the microbial consortium R2 (Figure 2C). The Shannon and Simpson indices in the *α* analysis of the three microbial consortia significant increased on Day 6 compared with that on the Day 3, indicating the presence of dominant genera on the day 3 and a significant increase in species diversity on the day 6 (Table 1). Additionally, The PCA analysis results for the three microbial consortia indicated clear differences in the composition of samples between the day 3 and day 6, with significant changes in community composition occurring during the lignin degradation process (Figure 3). The observed negative correlation between *Pseudomonas* and *Achromobacter* may be indicative of successional dynamics within the consortium. We propose a model in which these taxa dominate at different phases of lignin degradation, potentially driven by shifts in substrate composition or metabolic byproducts. This temporal niche partitioning, where one genus declines as the other proliferates, could account for the inverse abundance patterns. At the same time, because of two dominant genera, *Brucella* and *Sphingobacterium*, in microbial consortium R0 synergize with *Pseudomonas* in degrading lignin, microbial consortium R0 has the highest degradation efficiency among the three microbial consortia. The consistent dominance of *Pseudomonas* across all three consortia reflects strong functional selection under our lignin-enrichment conditions, despite their distinct environmental origins. This suggests a convergence toward its key catabolic role in lignin degradation, independent of the initial inoculum source (Xu et al., 2021).

**Table 1:** Statistical table of *α* diversity index of three microbial consortia.

Consortium	Shannon			Simpson		
	Day 3	Day 6	*p* value	Day 3	Day 6	*p* value
R0	0.79058	1.224334	0.0039	0.339786	0.557071	0.0039
R1	1.461754	1.800702	0.0039	0.461128	0.599113	0.016
R2	1.417295	1.791557	0.0039	0.478378	0.665268	0.0039

The Shannon and Simpson indices were averaged.

### 3.3 Annotation results of lignin-degrading enzymes and prediction of degradation pathways

The metagenome was annotated by Cazy annotation and KEGG annotation to analyze the AA (Auxiliary Activities) family genes and possible metabolic pathways related to lignin degradation.

**Figure 4:**
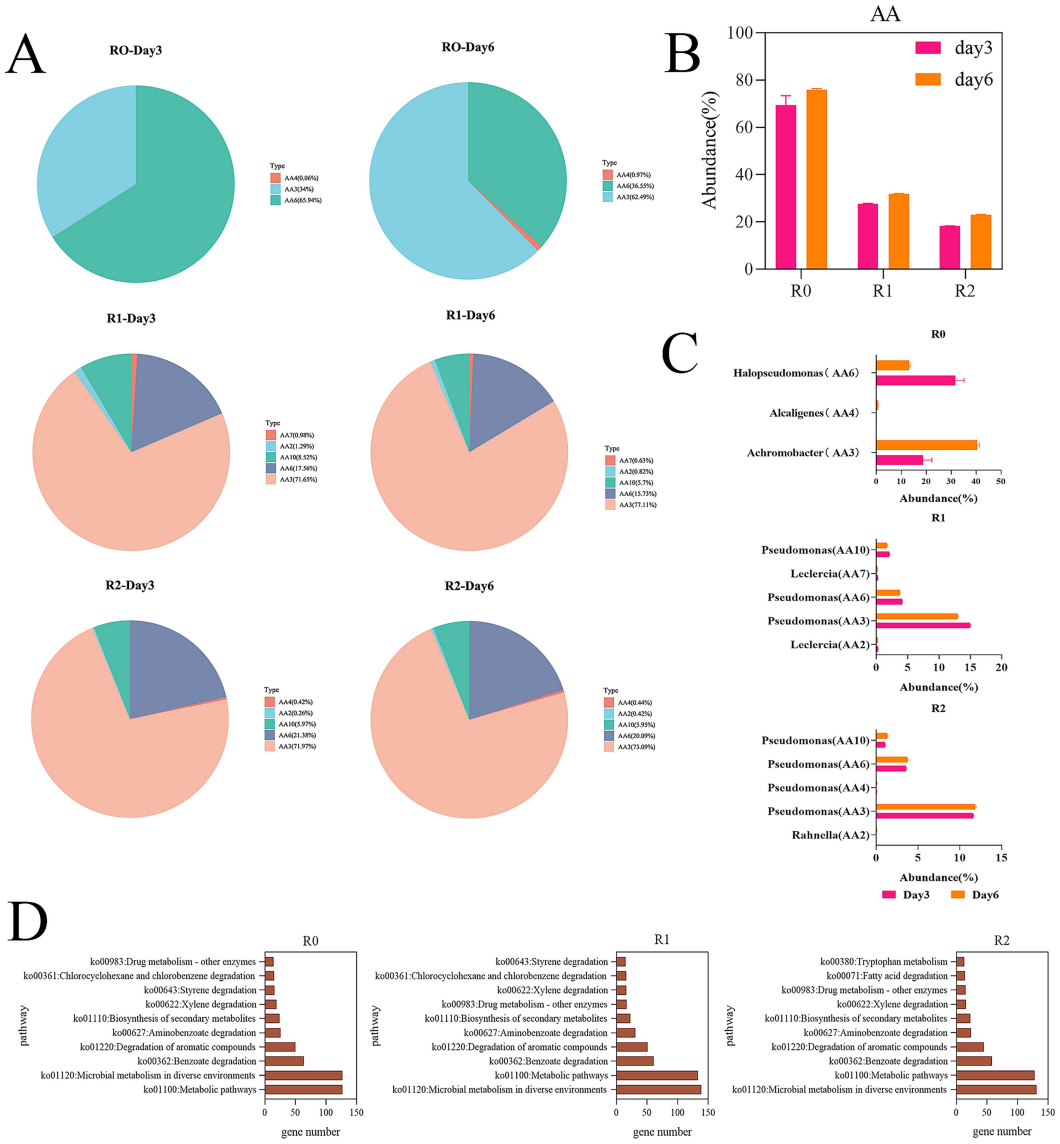
Analysis of key gene modules and statistics of metabolic pathways. **(A)** Results of the species abundance corresponding to the AA (Auxiliary Activities) family genes in the three microbial consortia. **(B)** Proportion of AA family genes in carbohydrate annotation enzymes in the three microbial consortia. **(C)** The genera with the highest percentage of AA family gene abundance in carbohydrate annotation enzymes in each of the three microbial consortia. **(D)** Statistics on the number of genes in the tertiary pathways for the metabolism of exogenous substances in the three microbial consortia.

Lignin degradation co-oxidoreductase family genes (AAs) are key genes in the lignin degradation process (Wang et al., 2024). AA3 is composed of FAD-dependent GMC oxidoreductase (Levasseur et al., 2013). AA2 family contains class II lignin-modified peroxidases, AA4 family contains vanillyl alcohol oxidase (VAO), AA6 family contains 1,4-benzoquinone reductase, AA7 family contains glucooligosaccharide oxidases, AA10 family contains copper-dependent lytic polysaccharide monooxygenase (Wang et al., 2024). Among them, manganese peroxidase of the AA2 family can act on the degradation of phenolic compounds. Non-phenolic compounds are degraded by lignin peroxidases from the AA2 family, multifunctional peroxidases from the AA3, AA4, AA6, AA7, and AA10 families, and lignin degradation auxiliary enzymes (Wang et al., 2024). In our study, the R0 microbial consortium contained AA3, AA4, and AA6 family genes. The R1 microbial consortium contains AA2, AA3, AA6, AA7, and AA10 family genes. R2 contains AA2, AA3, AA4, AA6, and AA10 family genes (Figure 4A). In our study, the abundance of AA family genes in all three microbial consortia increased on the day 6. The R0 microbial consortium had the highest abundance of AA family genes, which is consistent with the finding that R0 has the highest degradation efficiency (Figure 4B). On the day 6, the abundance of AA6 family genes decreased and AA3 family genes increased in all three microbial consortia. The analysis is related to the refractory degradation of benzoquinones. in the early stage of lignin degradation, there were more benzoquinones, and in the late stage, the refractory substances had been consumed, leading to a more intense redox reaction. In addition, the abundances occupied by the genera containing each series of AA family genes in the microbial consortia were ranked, and the genera with the highest abundance share of each series were selected (Figure 4C). We found that among the AA family gene series with the largest abundance.

*Halopseudomonas* and *Achromobacter* in microbial consortium R0 occupied greater abundance of AA family genes, and the other two microbial consortia occupying greater abundance of AA family genes were all *Pseudomonas*, so the key genera in the three microbial consortia were *Halopseudomonas* and *Achromobacter* in microbial consortium R0, and *Pseudomonas* in microbial consortia R1 and R2. In this study, we performed both 16S rRNA amplicon sequencing and shotgun metagenomics sequencing. For taxonomic annotation, we relied primarily on the 16S data due to its higher accuracy and resolution, which are based on rRNA-specific databases such as SILVA. In contrast, taxonomic assignments from metagenomics data were based on the general NR protein database, which provides lower resolution and cannot be directly matched to the 16S-based results on a per-sample basis. Although there are discrepancies in the annotation results, both consistently indicate that the two genera belong to the Pseudomonadaceae family at the family level, both 16S rRNA and metagenomic data consistently highlight members of the Pseudomonadaceae family as keystone taxa carrying AA genes across all consortia, with *Achromobacter* (a member of the Alcaligenaceae family) being a particularly important genus in R0. The exact genus-level identity of the key Pseudomonadaceae members may vary between analytical techniques, but their functional role in lignin degradation is strongly supported.

In order to find the key degradation pathways, we further performed KEGG annotation on the genes to identify the key degradation pathways. In this study, lignin, as the sole carbon source, was metabolized and utilized by the microorganisms in the form of exogenous substances during the metabolism process, therefore, we mined the key tertiary pathways under the exogenous substance metabolism secondary pathways, and then found the key lignin degradation pathways. According to the number of genes in the KEGG annotation results, the tertiary pathways under the metabolic pathways of exogenous substances were ranked. The top five tertiary metabolic pathways were Metabolic pathways, Microbial metabolism in diverse environments, benzoate degradation, degradation of aromatic compounds, and aminobenzoate degradation. The enrichment of these five pathways may be related to the presence of more benzene ring structures in the intermediate degradation products of lignin (Figure 4D). Therefore, we will analyze the possible degradation pathways of lignin in microbial consortium from the three pathways. In the three pathways, the aromatic substances in the lignin metabolites first go through the aromatic compound metabolism pathway and then part of them will enter the catechol metabolism pathway, followed by the *β*-ketoadipic acid metabolism pathway. The remaining aromatic metabolites are converted to benzoates and aminobenzoates, which subsequently enter the benzoate and aminobenzoate metabolic pathways, respectively. Following deamination of the aminobenzoates, both types of compounds converge into the protocatechuate pathway. The resulting intermediates then feed into the β-ketoadipate pathway, ultimately leading to entry into the tricarboxylic acid (TCA) cycle. The dominance of *Pseudomonas* in our study aligns with its known metabolic versatility. This is exemplified by *Pseudomonas putida* KT2440, which can degrade various benzoates and aminobenzoates (Jimenez et al., 2002), supporting the potential involvement of our *Pseudomonas* population in metabolizing aromatic compounds.

### 3.4 Analysis of lignin degradation metabolomics data

**Figure 5:**
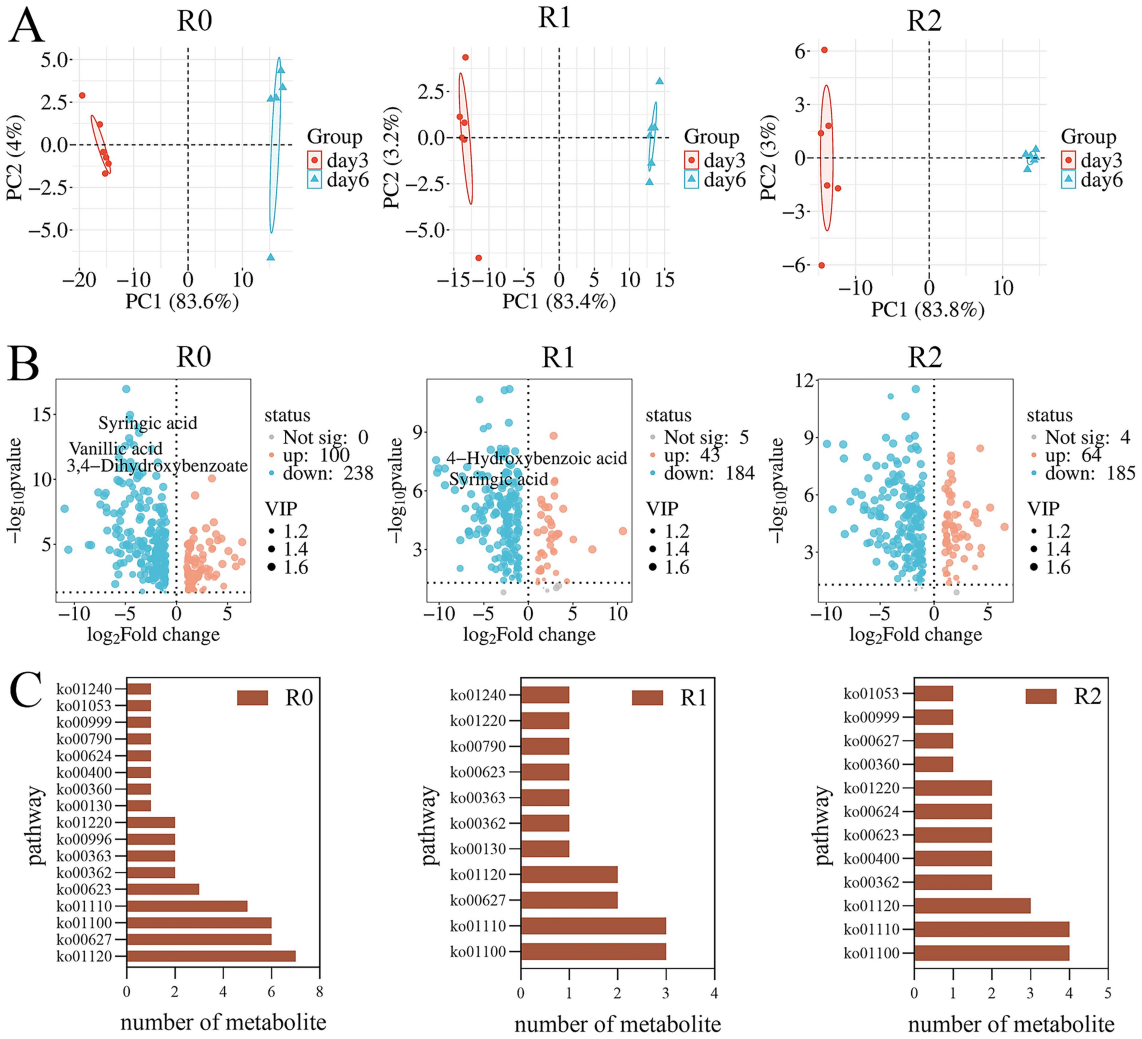
Metabolomic analysis. **(A)** PCA analysis of differential metabolites. **(B)** Differential metabolite volcano map. **(C)** Summary of differential metabolites KEGG-annotated metabolic pathways.

The metabolites of the three microbial consortia on day 3 and day 6 were determined with LC–MS. Metabolites exhibiting variable importance in projection (VIP) scores greater than 1.0, along with those demonstrating statistically significant contributions, were selected for PCA (principal component analysis) (Figure 5A) and illustrated in volcano plots (Figure 5B). PCA analysis indicated significant differences in the degradation products of the three microbial consortia between day 3 and day 6. Volcano plots illustrate the differential metabolites identified across the microbial consortia, a total of 338 metabolites were detected in R0, 232 in R1, and 253 in R2 (Table 2). Many other lignin metabolites were also detected in the three microbial consortia. Specifically, the R0 microbial consortium was found to contain Vanillin, 3,4-Dihydroxybenzoate, 2-Hydroxycinnamic acid, Ferulic acid, 3,4-Dimethoxybenzaldehyde, 4-Hydroxybenzaldehyde, Phenol, 3,4-Dihydroxybenzaldehyde and 3-Hydroxybenzoic acid. The lignin degradation-related substances of R1 microbial consortium contains p-Coumaraldehyde, methyl vanillate, Eudesmic acid, Phenol, Cinnamaldehyde, Myristicin and 4-Formyl-2-methoxyphenyl. The lignin degradation-related substances of R2 microbial consortium contains 3,4-Dimethoxybenzaldehyde, isopropyl 4-hydroxybenzoate, and Sinapyl alcohol, Cinnamic acid, Phenol, Cinnamaldehyde, and 3,4-Dimethylbenzoic acid (Table 2).

**Table 2:** Statistics of intermediate products of lignin degradation.

Category	Compounds	Belongs to the microbial consortium
Alcohol	Sinapyl alcohol	R2
Acid	Syringic acid	R0, R1
	Vanillic acid	R0
	Cinnamic acid	R2
	2-Hydroxycinnamic acid	R0, R2
	Ferulic acid	R0
	Eudesmic acid	R1
	3,4-Dimethylbenzoic acid	R2
	3,4-Dihydroxybenzoate	R0, R2
	4-Hydroxybenzoic acid	R0, R1
	3-Hydroxybenzoic acid	R2
Aldehyde	p-Coumaraldehyde	R1
	Cinnamaldehyde	R1, R2
	Vanillin	R0
	3,4-Dimethoxybenzaldehyde	R0, R2
	3,4-Dihydroxybenzaldehyde	R0
	4-Hydroxybenzaldehyde	R0
Ester	Methyl vanillate	R1
	Isopropyl 4-hydroxybenzoate	R2
	Methyl 2,5-dihydroxycinnamate	R2
Phenol	Phenol	R1, R2

Following depolymerization, the majority of lignin-derived intermediate metabolites are ultimately converted into benzoates, which are eventually degraded via the benzoate metabolic pathway. Previous reports have also shown that benzoates are key intermediates in the lignin degradation process (Ley et al., 2023). The metabolites that finally enter the metabolic pathway of benzoates are in the form of 4-hydroxybenzoic acid and 3,4-dihydroxybenzoic acid (Niu et al., 2021). Among them, the signature intermediate metabolites in R0 microbial consortium are vanillic acid, Syringic acid and 4-hydroxybenzoic acid. R1 contains 4-hydroxybenzoic acid and Syringic acid. However, no intermediates in the protocatechuic acid metabolic pathway were found in the metabolites of the R2 microbial consortium, presumably due to the slower material conversion process and slower protocatechuic acid metabolism in the R2 microbial consortium. This also reconfirms that the degradation rate of microbial consortium R2 is the lowest among the three microbial consortia. It was found by KEGG annotation of the metabolites that our lignin degradation intermediates are mainly annotated into aminobenzoate and benzoate metabolic pathways (Figure 5C), The enrichment of these pathways in the metagenomic functional profile further indicates a central role of aminobenzoic acid and benzoate metabolic pathways in the metabolic processes of the microbial consortia.

### 3.5 Mapping lignin degradation pathways

**Figure 6:**
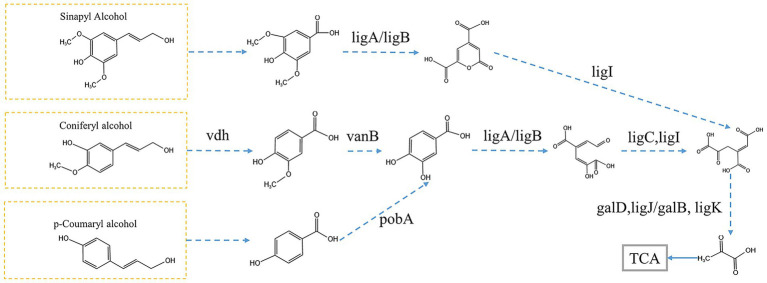
prediction of lignin degradation pathways. Enzyme names: *vdh* (vanillin dehydrogenase), *ligA*/*ligB* (protocatechuate 4,5-dioxygenase, alpha chain), *vanB* (vanillate monooxygenase), *pobA* (p-hydroxybenzoate 3-monooxygenase), *ligC* (2-hydroxy-4-carboxymuconate semialdehyde hemiacetal dehydrogenase), *ligI* (2-pyrone-4,6-dicarboxylate lactonase), *galD* (4-oxalomesaconate tautomerase), *ligJ*/*galB* (4-oxalmesaconate hydratase), *ligK* (4-hydroxy-4-methyl-2-oxoglutarate aldolase).

Based on the five pathways annotated by metagenomics KEGG, we chose three pathways including benzoate degradation, degradation of aromatic compounds, and aminobenzoate degradation for the analysis of lignin metabolic pathways. We performed statistical analysis of genes in these three pathways and compared the significant of differences genes in the KEGG pathway to find a complete degradation pathway (*p* < 0.05) (Figure 6). Through our analysis we found a complete protocatechuic acid degradation pathway present in the microbial consortia R0 and R1. Subsequent integrated analysis of metabolomics data was corroborated by metagenomic findings. As a core intermediate in lignin degradation, the abundance of protocatechuic acid is highly correlated with lignin content (Johnson et al., 2016). Consequently, the lack of a functional protocatechuic acid degradation pathway in Consortium R2 may underlie its observed sluggish degradation kinetics. Lignin could been depolymerized into three key intermediate compounds: Syringic acid, vanillic acid, and p-hydroxybenzoic acid (Ley et al., 2023). We found syringic acid in the microbial consortium R0 and R1, while R0 also contained vanillic acid. The highest number of key metabolites also indicates most lignin degradation pathways in R0, which corresponds to the highest degradation efficiency of R0 microbial consortium. It has been shown that Syringic acid and vanillic acid are degraded via the protocatechuic acid degradation pathway (Jimenez et al., 2002; Mueller et al., 2022). This is consistent with the results of the metagenomics analysis.

P-Hydroxybenzoic acid and vanillic acid can be converted into 3,4-dihydroxybenzoic acid directly. Meanwhile, we found that Syringic acid can be converted into 4-oxalomesaconate, which integrates into the Protocatechuic acid (3,4-dihydroxybenzoic acid) metabolic pathway and ultimately enters the TCA cycle. This metabolic pathway involves the genes of Protocatechuic acid by *ligA*/*ligB*, *ligC*, and *ligI* genes, leading to the formation of 4-oxalomesaconate, which is then regulated by *galD*, *ligJ*/*galB*, and *ligK* to form pyruvate, subsequently entering the TCA cycle to complete degradation (Figure 6). All of these genes are found in the differential genes identified in both microbial consortia R0 and R1. We analyzed the corresponding genera of this part of the gene from the metagenomics data, in which the combination of the genera *Achromobacter* and *Alcaligenes* in microbial consortium R0 can complete the protocatechuic acid degradation pathway, and the combination of *Pseudomonas* and *Delftia* in microbial consortium R1 can complete the protocatechuic acid degradation pathway (Table 3). In the 16S rRNA analysis results we also found the dominant genera *Achromobacter* in R0 and the dominant genera *Pseudomonas* in microbial consortium R1. Therefore, we suggest that *Alcaligenes* and *Delftia* play a synergistic degradation role for the dominant genera during lignin degradation, and we hypothesize that the protocatechuic acid degradation pathway is the core degradation pathway in the lignin degradation system in which *Pseudomonas* is the dominant genera, and that the change in protocatechuic acid can be used as an indicator to evaluate the efficiency of lignin degradation. It should be noted that this study utilized alkali lignin as a model substrate. To further validate the practical degradation capability of the microbial consortia, future work will employ crop straw, a more complex and natural lignocellulosic material. Subsequently, Fourier transform infrared (FTIR) spectroscopy and scanning electron microscopy (SEM) will be utilized to characterize the structural and chemical modifications of the straw following microbial treatment (Jain et al., 2023).

**Table 3:** Summary of protocatechuic acid degradation pathway genes and related microorganisms.

Gene	Genera of R0	Genera of R1
*vdh*	*Alcaligenes*	*Pseudomonas*
*vanB*	*Alcaligenes*	*Pseudomonas*
		*Delftia*
		*Leclercia*
*pobA*	*Brucella*	*Pseudomonas*
	*Alcaligenes*	*Delftia*
	*Achromobacter*	*Agrobacterium*
	*Bosea*	*Achromobacter*
		*Stutzerimonas*
		*Neorhizobium*
		*Klebsiella*
*ligA*	*Mesopusillimonas*	*Delftia*
	*Alcaligenes*	
*ligB*	*Pigmentiphaga*	*Delftia*
	*Alcaligenes*	*Delftia*
	unclassified_Betaproteobacteria_genus	*Pseudomonas*
	*Brucella*	*Agrobacterium*
*ligC*	*Acidovorax*	*Eoetvoesia*
	*Alcaligenes*	
	*Mesopusillimonas*	
*ligI*	*Xylophilus*	*Delftia*
	*Alcaligenes*	
	*Mesopusillimonas*	
*ligJ*	*Paracandidimonas*	*Delftia*
	*Comamonas*	*Paracandidimonas*
	*Alcaligenes*	
	*Pigmentiphaga*	
	*Mesopusillimonas*	
*ligK*	*Achromobacter*	*Delftia*
	*Acidovorax*	*Agrobacterium*
	*Alcaligenes*	*Pseudomonas*
	*Mesopusillimonas*	*Variovorax*
	*Pigmentiphaga*	*Pseudorhizobium*
	*Bosea*	
*galB*	*Pusillimonas*	*Stutzerimonas*
	*Methylobacterium*	*Pseudomonas*
	*Achromobacter*	*Pseudorhizobium*
	*Pigmentiphaga*	*Pusillimonas*
		*Pollutimonas*
		*Eoetvoesia*
*galD*	*Alcaligenes*	*Delftia*
	*Bosea*	*Stutzerimonas*
		*Shinella*
		*Pseudorhizobium*
		*Sphingobium*

## 4 Conclusion

The degradation of lignin is a challenging task. All three microbial consortia had stable lignin degradation ability, and the R0 microbial consortium had the highest degradation efficiency. The two dominant genera for each of the three bacterial lineages R0, R1, R2 are Pseudomonas and Achromobacter, Pseudomonas and Achromobacter, Pseudomonas and Sphingobacterium, respectively. The gene annotation from the metagenome and KEGG annotation results for lignin degradation intermediates indicate that the main degradation pathways of the three microbial consortia are the aminobenzoic acid and benzoic acid metabolism pathways. The abundance of AA family genes in the R0 microbial consortium accounted for the highest proportion of carbohydrate active enzymes with highest degradation efficiency of microbial consortium R0. Protocatechuic acid is a central intermediate in the degradation of lignin. The protocatechuic acid degradation pathway was fully annotated in R0 and R1 microbial consortia. The Achromobacter and Alcaligenes in R0 microbial consortium can work together to achieve this process, as can the Pseudomonas and Delftia in the R1 microbial consortium. The protocatechuic acid degradation pathway is the key degradation pathway in the lignin degradation process with Pseudomonas as the dominant genera, and protocatechuic acid content can be used as an indicator of lignin degradation. Furthermore, the mechanistic analysis revealed that Consortium R0 possessed distinct traits associated with its superior performance, including the highest abundance of AA enzymes, a unique microbial composition, and a complete protocatechuic acid degradation pathway. This study enriches the efficient and stable degradation of lignin by microbial consortia, and also provides theoretical backing for the mechanisms of lignin degradation.

## Data Availability

The datasets presented in this study can be found in online repositories. The names of the repository/repositories and accession number(s) can be found below: https://www.ncbi.nlm.nih.gov/, PRJNA1327111.
